# The Role for Epigenetic Modifications in Pain and Analgesia Response

**DOI:** 10.1155/2013/961493

**Published:** 2013-10-20

**Authors:** Sherrie Lessans, Susan G. Dorsey

**Affiliations:** ^1^School of Nursing, University of Maryland, Baltimore, USA; ^2^Program in Neuroscience, University of Maryland, Baltimore, USA

## Abstract

Pain remains a poorly understood and managed symptom. A limited mechanistic understanding of interindividual differences in pain and analgesia response shapes current approaches to assessment and treatment. Opportunities exist to improve pain care through increased understanding of how dynamic epigenomic remodeling shapes injury, illness, pain, and treatment response. Tightly regulated alterations of the DNA-histone chromatin complex enable cells to control transcription, replication, gene expression, and protein production. Pathological alterations to chromatin shape the ability of the cell to respond to physiologic and environmental cues leading to disease and reduced treatment effectiveness. This review provides an overview of critical epigenetic processes shaping pathology and pain, highlights current research support for the role of epigenomic modification in the development of chronic pain, and summarizes the therapeutic potential to alter epigenetic processes to improve health outcomes.

## 1. Introduction

Pain is the number one reason patients consult a health care provider in the United States, with one in every three emergency room patients and more than 60% of all primary care patients listing pain as their chief complaint [1]. Chronic pain, including migraine headaches and low back pain, affects more than 250 million Americans and nearly 10% of the world's population. The incremental health care and societal costs of undermanaged pain range from $560 to $635 billion annually in the United States, including lost worker productivity and the impact of addiction, with another $900 billion worldwide [1–4]. Pain, however, remains a poorly understood symptom. No person experiences pain like any other and even the same person may experience pain in different ways, at different times, and under different circumstances challenging both assessment and treatment. Without a clear understanding and consensus as to the mechanisms underlying these differences, nurses are limited in their ability to develop an evidence-based intervention science to guide symptom management. The incorporation of genetic approaches into nursing and multidisciplinary research has been one of the most significant research developments in the last 10 years, providing new and promising opportunities to understand interindividual differences in pain and therapeutic response. 

Pain genetics is a broad term that describes both classic Mendelian techniques used to identify inherited variation in pain sensitivity and analgesic response as well as newer gene-level DNA and RNA sequence measurement sciences. While these techniques and approaches are not novel, their application to understanding pain and pain management has provided new mechanistic insights and treatment possibilities. Genome-wide association studies (GWAS) and focused candidate gene association studies (CGAS) have identified more than 350 genes that are relevant in both clinical and experimental pain, with identification of many hundreds more pain and analgesia regulating genes [5, 6]. Polymorphisms of pain-relevant genes identified through genetic linkage mapping suggest that heritable genetic factors play a role in many pain states including menstrual, migraine, and musculoskeletal pain and help to explain some interindividual differences in pain and analgesic response [7–12]. Genetic correlation studies in selective bred mouse strains have identified common sets of genes associated with phenotypic clusters of pain traits, suggesting different forms of nociception and hypersensitivity that represent genetically distinct pain modalities [5, 8, 13–15]. Mechanical nociception is likely mechanistically and genetically distinct from both thermal nociception and the nociceptive response to noxious chemical stimuli, with each modality also likely having unique analgesic responsiveness. Advances in animal modeling linking genetic variability to differences in pain and analgesic response have successfully translated to many testable gene-associated hypotheses in human pain studies [5, 9].

However, not all attempts to isolate the effects of DNA variance have been successful. Many complex pain conditions including rheumatoid and osteoarthritis as well as fibromyalgia and neuropathic pain do not link to heritable factors. Genetic approaches also have not been generally useful in identifying factors that shape the trajectory of pain symptoms distinct from the pathophysiology of disease [15–18]. Although variability in techniques and experimental methods across laboratories has contributed to difficulties in replicating some research findings, evidence suggests that there are factors beyond biological variability across populations or pain states that shape the pain experience. A new focus on epigenetic mechanisms has highlighted the role that highly orchestrated remodeling of transcription and translation processes play in altering genomic structure and function without any change in the basic nucleotide sequence in DNA [18, 19]. In this review, we introduce fundamental concepts of epigenetics, highlighting current and future prospects for developing a richer understanding of human pain as well as more effective pain management. 

## 2. Epigenetic Mechanisms: Chromatin Remodeling, Modification, and Gene Expression

The human genome contains more than 6 billion individual base pairs of amino acids packaged onto 23 paired chromosomes, supporting more than 30,000 regionally localized genes. These transcription and regulatory sequences of nucleic acid molecules are responsible for coding the instructions that make proteins and other critical cell products. Compacting the nearly 2 meters of chromosomal DNA into the relatively small nucleus inside each cell, while continuing to support transcription, replication, and ultimately gene expression, requires a series of highly coordinated packaging processes that serve to temporally and functionally control access to DNA throughout the cell cycle [19–21]. Histones are the chief compacting proteins within the nucleus. These positively charged proteins develop tight covalent bonds with the negatively charged proteins along the backbone of DNA creating spooled DNA-histone complexes called chromatin. Chromatin organizes as repeating structural and functional groupings of eight histones surrounded by short segments of spooled DNA called nucleosomes. This “bead on a string” chromatin structure opens segments of DNA, facilitating transcription and replication of DNA by allowing ready access to RNA and DNA polymerases as well as other transcription accessory proteins. Chromatin can also condense around multiple histones into short, thick, coiled nucleosome-dense fibers; this tightly compact chromatin structure prevents access to the DNA, effectively silencing gene expression [22–24]. It is this functionally relevant regulation of gene expression through dynamic remodeling and modifications to chromatin that defines epigenetics [19–21]. Tightly regulated purposeful alterations in chromatin conformation enable cells to control transcription, replication, protein production, and ultimately survival. Pathological alterations in chromatin conformation adversely affect the ability of the cell to respond to physiologic and environmental cues and are linked to disease and reduced treatment response. The promise in epigenetics lies in identifying the temporal ordering of chromatin conformational changes linked to pathology, then therapeutically leveraging the transient and often reversible nature of epigenetic processes to interrupt or otherwise influence a health outcome [20, 21, 25–27].

Cells have evolved considerable diversity for altering the way DNA compacts around histone proteins, providing an almost unlimited ability to control and shape DNA readout. Chromatin remodeling processes include those that specifically target the genomic DNA within the chromatin structure, specifically and principally DNA methylation. Other processes target the histone proteins, including processes that add or remove methyl, acetyl, and phosphate groups. Still other processes shape the actions of small regulatory noncoding and gene-silencing RNAs. DNA methylation involves the addition of a methyl group to a cytosine in a DNA dinucleotide; this modifies the covalent bonding between DNA and histones providing a stabilizing effect on gene expression [28, 29]. Entire genomic regions in the DNA have been identified where cytosine and guanine appear next to each other in repeating sequence, held together by phosphodiester bonds. The methylation status of cytosine in these CpG islands exerts a robust influence on gene expression. Transient methylation of CpG sites in coding regions of genes will temporally suppress gene expression, while unmethylated CpG sites in promoter regions will increase gene expression [20, 21]. Although methylation of DNA can function to suppress harmful sequences of DNA that have been integrated into the genome over many generations, DNA methylation has also been implicated in the development of many cancers. Methylated DNA has been demonstrated to disrupt the binding of transcriptional proteins as well as the recruitment of other remodeling proteins, effectively silencing tumor suppressor genes allowing rapid and often unregulated tumor growth [30, 31]. 

The linear structure of histone proteins includes sequences of amino acids that have been translated from messenger RNA and then folded and held together by both weak and strong covalent bonds. These amino acid sequences or residues can undergo a wide variety of posttranslational enzymatic modifications that also influence how DNA compacts around the histone core. Similar to methylation in the DNA dinucleotide, the addition of a methyl group to a histone protein generally inhibits gene expression, while the addition of an acetyl group into a histone protein generally loosens the interaction of DNA and histones favoring gene transcription [20, 22, 24, 25]. The orchestrated formation and disruption of chromatin that controls transcription also include ways to rapidly and dynamically demethylate and deacetylate protein structures, add or remove phosphate groups, and to recruit a wide range of modifier proteins that alter the covalent connections to other proteins in the cell [19, 20].

## 3. Epigenetic Mechanisms Shaping Pain and Analgesia Response

Chromatin modifications and remodeling are most pronounced when cells experience rapid environmental changes and chemical stress [32–35]. Many cell types utilize a wide range of epigenetic mechanisms to withstand and respond to insult. Mature neurons, however, with very low turnover and regeneration rates, likely owe their long-term survival across a life time of environmental misfortune to a broad and comprehensive epigenetic response to cellular stress [33, 34]. Where most pain research has traditionally focused on understanding the underlying genomics and pharmacogenetics of injury, inflammation, and pain, epigenetics provides a new paradigm with which to explore the plasticity of the nervous system [5, 36–38]. All along the nociceptive pathway, from periphery to cortex, a wide range of molecular mechanisms exist to either facilitate or inhibit the processing of pain messages. Acute pain usually follows localized injury and inflammation, sensitizing both the peripheral and central nervous system, and prompting tissue protective withdrawal responses. Sensitized spinal and brain nerve cells respond to persistent afferent input by releasing both pro- and antinociceptive molecules, mediating and moderating pain responses, and in the process altering their synaptic relationships with adjacent nerve cells. Some synaptic connections will quiet or die back, some new synapses appear, and some abnormal synaptic connections will form, changing the balance of excitation and inhibition in pain processing. Researchers describe the formation of abnormal or pathologic connections as a form of “cellular memory” explaining why pain may linger after all objective measures indicate tissues that have healed [39–41]. After decades of pain research, however, it still is not clear why one patient develops persistent or chronic pain and a second patient with a very similar insult will not. Epigenetic modifications may well represent the physiologic link between the injury state, the wider environment, and chronic pain, with impact apparent from the first moments of tissue insult.

Several critical “first responder” transcription factors, including NF-kB (nuclear factor k-light-chain-enhancer of activated B cells), c-Jun, c-Fos, and several hormone activated receptor proteins, serve as drivers for wide-ranging epigenetic responses to cellular stress. Present but inactive in many vascular, nerve, and immune cells, these transcription factors become activated in response to cell insult and are able to rapidly access chromosomal DNA as demethylation unspools chromatin structure to initiate production of a reparative cascade of inflammatory cytokines including TNF-*α* (tumor necrosis factor-alpha) as well as T-cell and B-cell regulating interleukins [42–44]. The nearly simultaneous dynamic remodeling of chromatin through the addition of methyl groups to DNA and the removal of acetyl groups from histone proteins regulates production of immune suppressing glucocorticoids, providing a critical check and balance to overactivation of immune responses [44–46]. Epigenetic changes to chromatin structure are similarly linked to suppression of pain inhibiting GABA (Gama-amino butyric acid) synthesis, changes in expression patterns of sodium and potassium channels driving afferent input into the spinal cord, and activity-dependent upregulation of pronociceptive brain-derived neurotrophin factor (BDNF) in the spinal cord, as well as functional regulation of mu opioid receptors, the principle receptor for endogenous endorphins, encephalin and as well as opioid analgesics [47–49]. 

## 4. Potential for Greater Mechanistic Understanding for Chronic Pain

Two of the most therapeutically intriguing insights arising from epigenetics research are suggestions that epigenetic mechanisms play a critical role in the transition from acute to chronic pain, and that a wide range of environmental factors across the lifespan serve as epigenetic primers for individual pain and analgesic response [5, 26, 38]. Evidence suggests that more than 1,000 genes in SDH (spinal dorsal horn) neurons are epigenetically regulated within the first minutes to hours following a peripheral nerve injury (40, 43, and 53). Often, these early modifications are followed by more sustained epigenetic processes shaping synaptic connectivity and formation of pathologic long-term pain. Sustained DNA methylation downstream from early effector transcription factors, for example, has been linked to an accelerated degeneration of vertebral disks in low-back pain in both animal models and human subjects. Sustained histone deacetylation has been identified as a factor driving long-lived C-fiber dysfunction, decreased responsiveness to morphine analgesia, and an upregulation of pronociceptive metabotropic glutamine receptors in animal nerve injury models [38, 48, 50–53].

Variable production of stress-induced glucocorticoids, variable response to exogenously administered steroidal anti-inflammatory agents, and even glucocorticoid resistance are all identified as the likely mechanisms responsible for autoimmune illness and pathologic chronic pain, with each linked to underlying epigenetic processes. Diverse and high individual methylation patterns are associated with alternative activation of promoter sites producing different sensitivities to glucocorticoids. These diverse methylation patterns have been shown to be associated with a number of environmental factors including diet, maternal care, and early life stressors. This provides compelling mechanistic evidence in support of long-observed linkages between early sexual or physical abuse, neonatal pain, previous injuries, and chronic pain later in life [45, 46]. Epigenetics processes also provide a mechanistic understanding of the phenomenon of opioid-induced hyperalgesia, with chronic opioid use reported to stimulate DNA methylation leading to upregulation of *µ*-opioid receptors and increased pain with continued opioid use [54]. 

## 5. The Therapeutic Potential in Blocking the Deacetylation of Histones

The critical role of deacetylation of histone proteins in shaping pain pathophysiology and analgesic response is highlighted in a series of experiments, where deacetylation has been pharmacologically inhibited [55–57]. Currently, there are at least eighteen known genes which code for histone deacetylases (HDACs), with differential expression patterns throughout the human nervous system [58]. Exogenous intrathecal administration of HDAC inhibitors results in attenuation of experimental inflammatory pain induced by complete Freund's adjuvant (CFA) and formalin in rodents [52–55]. HDAC inhibitors administered in central descending pain pathways result in decreased acetylation of the Gad65 (glutamate decarboxylase) and Gad67 promoters in rodent models of nerve injury. These enzymes normally catalyze the production of GABA resulting in enhanced GABA inhibition and reduced pain. The sustained hypoacetylated state of Gad promoters following nerve injury in rodents suggests that this may be a pathologic feature in chronic pain that can be overcome by blocking removal of acetyl groups from histone complexes [47]. Translating these mechanistic insights to promising human trials is beginning to show positive results. Valproic acid, long used to treat seizures, has been identified as a potent inhibitor of Class I and II histone deacetylases and is being used in a prophylaxis trial to treat migraine headache. Givinostat, a second HDAC inhibitor, is being tested in the treatment of an idiopathic form of juvenile arthritis [59–62].

While pharmacologic blockade or inhibition of acetyl group removal from histones is showing early promise, current approaches to therapeutically manage the methylation status of DNA present a more mixed picture of success. Glucosamine and L-methionine, for example, are endogenously produced molecules utilized by the body for biosynthesis of cartilage-repairing glycoproteins and glycosaminoglycans. Pharmaceutical grade versions of these molecules are both prescribed and taken over the counter as nutritional supplements for joint health and pain management for patients with osteoarthritis. Evidence suggests that these molecules also disrupt or alter methylation status of chromatin. To date, however, no well-designed studies report improved pain relief using these supplements over placebo controls [59–62]. More encouraging results are associated with the administration of folate, a B-vitamin given to pregnant women to reduce the risk of neural tube deficits. The widely administered supplement has also been demonstrated to serve as a critical cofactor for DNA methylation during pregnancy, with links to improved gastrointestinal health in adulthood [63–65]. The challenges involved with therapeutic manipulation of epigenetic processes are numerous. Currently, there is a lack of available agents with targeted specificity for any particular chromatin feature. Because the relationship between pathology and pain is complex and it is not clear whether epigenetic mechanisms represent the cause or the effect of pain states, it becomes difficult to know when it is best to disrupt epigenetic mechanisms to prevent pathology and pain [1, 32–34, 55].

One exciting growth area for symptom research, including pain and pain management, is evidence suggesting that the epigenetic state of chromatin interacts with and is critically shaped by context and environment. A new emphasis on how epigenetics may shape and be shaped by best practice and even moment-to-moment care decisions is likely to have a profound impact on the practice of both medicine and nursing, especially as it relates to critical event management. Neonatologists now recognize that micro- and macronutrition during pregnancy have an impact far beyond the early months of life, with nutritional support strategies epigenetically linked to later development of a wide range of adult illnesses including asthma, hypertension, colitis, and malignancies [63–65]. Critical care and anesthesia clinicians are beginning to describe the period before, during, and after the operative experience as the periotome, a period where “priming” of the genetic and epigenetic state can broadly influence biological results including hypoxic responses, depth of anesthesia, postoperative pain levels, and analgesia response [66]. The hope for the future would be to create a “prosurvival” phenotype through targeted perioperative epigenetic modification, with a more stable operative course, improved operative outcomes, and less postoperative pain. In a similar way, researchers that study trauma outcomes are exploring promising ways to modulate acetylation in the acute resuscitation phase to create an “anti-inflammatory” phenotype, lessening the effects of blood loss, shock, and pain [67]. Such promising research reinforces the premise that the nervous system has untapped capacity to respond to insult, inflammation, and injury. Health outcomes are critically shaped by epigenetic processes that are theoretically reversible and often transient, providing a new paradigm for developing more effective approaches and treatments to manage pain. 
